# A Rare Case of Cysticercosis Involving the Whole Spinal Canal

**DOI:** 10.1007/s11686-021-00486-1

**Published:** 2021-12-01

**Authors:** Xiaoyan Zheng, Fei Wang, Lei Wang, Xiaoli Li, Jingjing Li, Minjun Huang, Yang Zou

**Affiliations:** 1 Emergency and Critical Care Center, Beijing Friendship Hospital, Capital Medical University, Beijing, China; 2 Beijing Institute of Tropical Medicine, Beijing, China; 3 Beijing Key Laboratory for Research on Prevention and Treatment of Tropical Diseases, Beijing, China

**Keywords:** Cysticercosis, Neurocysticercosis, *Taenia solium*, Spinal cysticercosis

## Abstract

**Background:**

Cysticercosis is the commonest parasitic disease to affect the central nervous system (CNS). However, cysticercosis affecting the spine is extremely rare. We reported a rare case of cysticercosis involving the whole spinal canal in China.

**Case Presentation:**

A rare case of cysticercosis involving the entire spinal cord, in a 52-year-old Chinese man, was detected in 2021. Epidemiological investigation, clinical and etiological examination was performed.

**Conclusion:**

Since spinal cysticercosis is a rare but potentially life-threatening disease, clinicians should always consider the differential diagnosis of space-occupying lesions.

## Background

Cysticercosis is a major public health issue in most developing countries and is also becoming increasingly prevalent in developed countries [1]. Neurocysticercosis (NCC) occurs when the larval form of the *Taenia solium* (*T*. *solium*) invades the CNS. Spinal cysticercosis is rare, occurs in just 0.7–3% of NCC patients [2]. Here, we report a rare case of cysticercosis involving the entire spinal cord.

## Case Presentation

**Fig. 1:**
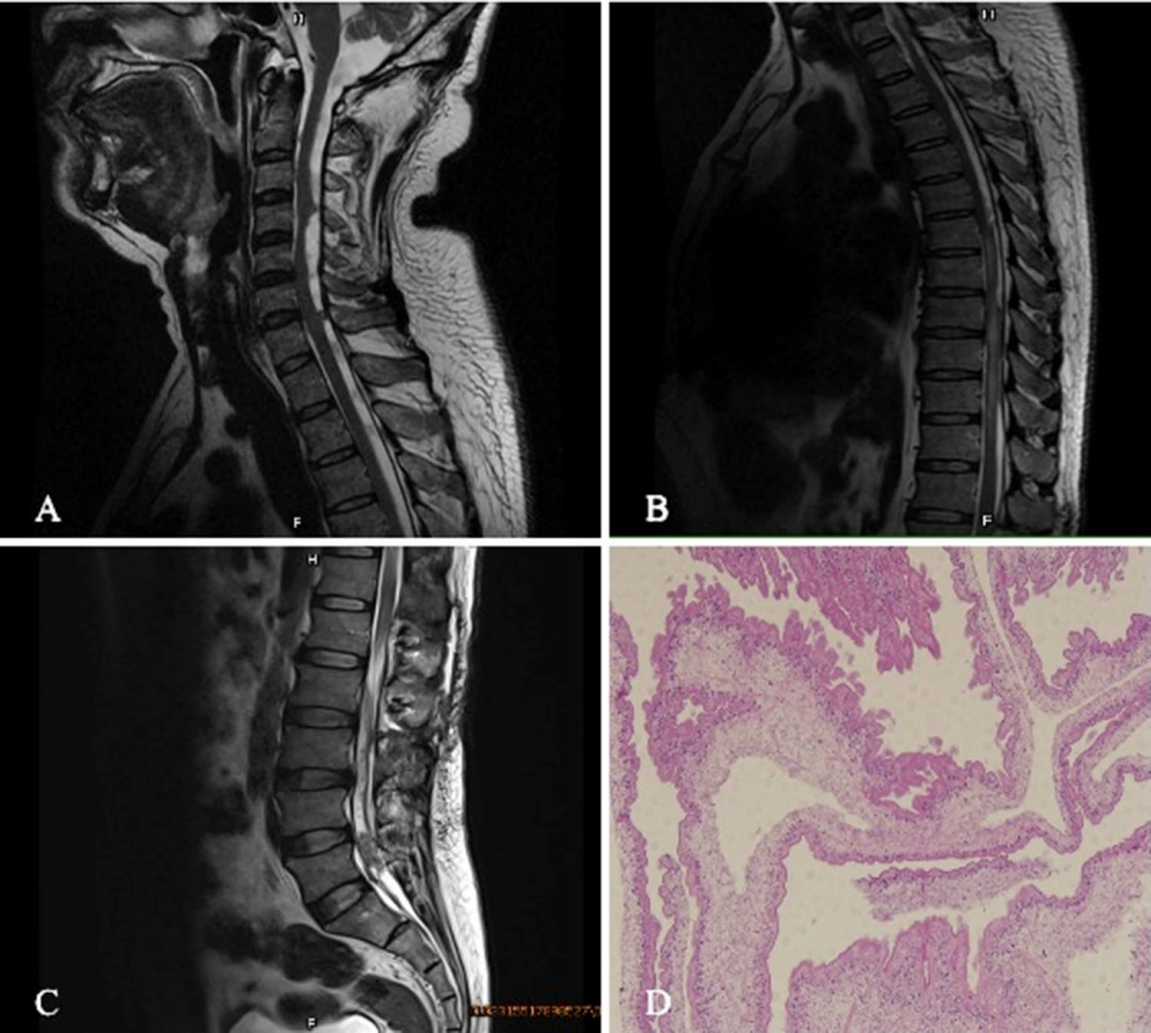
MRI of his spine showed that multiple cystic foci around the spinal cord (**A**, **B**, **C**). Hematoxylin and eosin staining indicated typical cysticercosis with an eosinophilic outer cuticle layer and a single-layered sub-cuticle cell (**D**)

A 52-year-old man felt numbness and pain in the waist for 9 months before coming to our hospital. Six months ago, the patient developed numbness in both lower limbs. He could walk, but he was unable to run, and he had headache occasionally. Three months ago, the patient had difficulty in urinating and defecating, and he was unable to walk. After 1 month of antituberculosis treatment and the removal of spinal tumor in other hospital, the symptoms were not improved. Therefore, he came to our hospital for further treatment. Neurological examination revealed spastic paraparesis with decreased motor power in both the lower limbs. Lumbar puncture was performed on admission, and the intracranial pressure was 140 mmH_2_O. Routine examination of cerebrospinal fluid: pale yellow, clear and transparent, no clot, negative Pandy test, white blood cells in cerebrospinal fluid were 16.0 × 10^6^/L (the normal range is 0–8.0 × 10^6^/L), red blood cells in cerebrospinal fluid were 0.0 × 10^6^/L, and white blood cell classification—mononuclear cells were 93% and multinuclear cells were 7%. Cerebrospinal fluid biochemistry: cerebrospinal fluid total protein (UCFP) 1034.17 mg/dl (the normal range is 15–45 mg/dl), cerebrospinal fluid potassium (K) 3.38 mmol/l (the normal range is 2.5–3.2 mmol/l), cerebrospinal fluid sodium (NA) 143.70 mmol/l (the normal range is 138–150 mmol/l), cerebrospinal fluid chlorine (CL) 114.90 mmol/l (the normal range is 120–132 mmol/l), cerebrospinal fluid carbon dioxide (CO2) 23.50 mmol/l (the normal range is 20–29 mmol/l), cerebrospinal fluid glucose (Glu) 3.49 mmol/l (the normal range is 2.24–3.92 mmol/l). The IgG antibodies of cysticercosis in both blood and cerebrospinal fluid were positive. MRI of his brain showed no obvious abnormality. MRI of his cervical spine showed the multiple cystic foci around the spinal cord at and below the level of axis vertebral body, small ring abnormal signal at the level of C6–7 intervertebral space, considering infectious disease (Fig. 1A). MRI of his thoracic spine showed long strip-shaped abnormal signals around the spinal cord at the level of C7–T6, and strip-shaped and beaded abnormal signals around the spinal cord at the level of T6–11 (Fig. 1B). MRI of his lumbar spine showed that degenerative changes of lumbar spine, postoperative changes of lumbar 1 and 2 adnexal areas as well as long strip and multi-cystic abnormal signals in the spinal canal at the level of L2–5, which were consistent with the manifestations of infectious diseases (Fig. 1C). The pathological section of the patient’s spine mass indicated typical cysticercosis with an eosinophilic outer cuticle layer and a single-layered sub-cuticle cell (Fig. 1D).

The patient was from Heilongjiang Province of China. He liked to eat roast meat and lettuce dipping sauce all the year round. He denied the history of defecation parasites or subcutaneous masses.

According to the patient’s symptoms, epidemiological history, auxiliary examinations and pathological results, he was diagnosed as cysticercosis. The lesion involves the entire spinal cord. After 10 days of treatment with albendazole 20 mg/kg/day for insecticidal treatment, dexamethasone for anti-inflammatory treatment, and cobamamide adenosine for nutritional nerve therapy, the patient’s paralysis of both lower limbs improved significantly, and the lower limbs were able to move slightly on the bed. The patient was followed up for 3 months, and the clinical symptoms did not deteriorate. This patient will continue to be treated and followed up in our hospital in the future.

## Discussion

Cysticercosis is a zoonotic disease caused by the larvae of *T. solium* [1, 5]. After people ingest the *T. solium* eggs shed in the stool of a human tapeworm carrier, embryos are released by the effect of bile and gastric enzymes,then, they invade the bowel wall, and disseminate hematogenously to striated muscles, liver, brain, and/or other tissues, so cysticercosis can occur in subcutaneous muscles, eyes, the CNS, spinal cord and heart. It infects almost 50 million people in the world and 3–6% population in endemic regions such as Central and South America, Africa, Eastern Europe, and the Indian subcontinent [7]. It is an important public health problem in many developing countries, also in some developed ones because of migration and tourism. It has been a great threat to people’s health in China [14]. The western parts of China are major local endemic areas now, because traditional husbandry, consumption of raw pork and poor sanitation are common there [17]. A survey of 484,210 participants from 31 provinces in China from 2014 to 2015 found 1752 positive taeniasis cases through microscopic examination [12].

When the larvae of the *T. solium* invade the CNS, NCC occurs. NCC includes parenchymal cysticercosis, leptomeningeal cysticercosis, intraventricular cysticercosis, and spinal cysticercosis according to the infected location. Spinal cysticercosis is rare and occurs only in 0.7–3% of the NCC patients [8]. Spinal cysticercosis includes four types (extradural cysticercosis, intradural cysticercosis, subarachnoid cysticercosis, and intramedullary cysticercosis) in location [11]. Intramedullary cysticercosis is rare, no more than 100 cases reported until now [16]. Spinal distribution is 34% in cervical, 44.5% in thoracic, 15.5% in lumbar and 6% in sacral regions [2]. In a review literature of spinal cysticercosis in 2016, cervical lesions, thoracic lesions, or multiple lesions were the main types [4]. Our patient was a rare case of cysticercosis involving the whole spinal canal in China, that has not been reported until now.

The detailed mechanism of NCC is unknown, and the cysticercus may invade the spinal cord through reaching the subarachnoid space from cerebral ventricles, retrograding blood flow by the vertebral and intervertebral veins, or migrating transpially. The symptoms of patients with spinal cysticercosis rely on the location and number of lesions, personal immune response, and the previous infection history. The main clinical manifestations are progressive weakness and myelopathy, paralysis, sensory disturbance, bladder incontinence, sometimes manifested as Brown-Séquard syndrome.

Diagnosis of NCC depends on epidemiological history, clinical manifestations, positive cysticercosis antibody of serum or cerebrospinal fluid by immunological examination, cystic lesions on CT or MRI, and pathological examination of tissues where the parasite was found [6]. CSF examination of patients with NCC often shows increased in lymphocytic cells or/and eosinophils, elevated protein, and normal/low glucose level. ELISA of CSF can be helpful for diagnosis of cysticercosis. The location and morphology of the cysts, the infection burden, the stage of cysts, and the surrounding inflammation can be revealed by CT and MRI. The scolex of cysticercus is rarely visible on CT or conventional MRI sequences, but can be found in apparent diffusion coefficient images and diffusion-weighted maps [3]. Pathological examination of diseased tissues to find parasites is the gold standard for diagnosis of cysticercosis.

Since spinal cysticercosis is a rare but potentially life-threatening disease, clinicians should always consider the differential diagnosis of space-occupying lesions. Spinal cysticercosis should be differentiated from the following diseases: they are congenital cysts, hydatid cysts, tuberculosis, autoimmune diseases, neoplasia, etc. They can be differentiated by epidemiological history, immunological antibody examination, CT or MRI and pathological examination.

Surgery can eliminate compress, particularly in patients with acute symptoms, and when the diagnosis of cysticercosis is unclear. Medical treatment of spinal cysticercosis contains combination of Albendazole and steroids. Albendazole can penetrate the blood–brain barrier and get a higher concentration in CSF, so it is the preferred parasiticides for spinal cysticercosis [9]. Steroids can reduce the acute inflammatory response when parasite dies and increase serum level of albendazole. After 10 days of treatment with albendazole 20 mg/kg/day for insecticidal treatment, dexamethasone for anti-inflammatory treatment, and cobamamide adenosine for nutritional nerve therapy, our patient’s symptoms were relieved greatly. The patient was very satisfied with our treatment.

## Conclusion

Spinal cysticercosis is a rare parasitic disease but can lead to serious neurological complications. Isolated intramedullary cysticercosis is extremely rare and no more than 100 cases have been reported in the world [13]. Cysticercosis should be taken into account in the diagnostic differentiation of spinal cord diseases. Since its imaging features like other spinal cord diseases, doctors may make misdiagnosis. Yoo M. reported that only 1 case was diagnosed as spinal cysticercosis among the 7 cases of spinal cysticercosis preoperatively [15]. It can be differentiated from other diseases by epidemiological history, immunological antibody examination, and pathological examination. We expect our case could draw everyone’s attention to spinal cysticercosis. If you encounter a similar situation, you can think of the disease as soon as possible to reduce misdiagnosis and mistreatment.

## Data Availability

The data supporting the conclusions of this article are included within the article. Mrs. Xiao-Yan Zheng is the contact person for availability of data and materials.

## Abbreviations

CNS: Central nervous system
NCC: Neurocysticercosis
*T. solium*: *Taenia solium*
